# Association of Fibrinolytic Potential and Risk of Mortality in Cancer Patients

**DOI:** 10.3390/cancers15174408

**Published:** 2023-09-03

**Authors:** Gabriele Silva Souza Gois, Silmara Aparecida Lima Montalvão, Thaizy Ramires Alencar Anhaia, Millene Evelyn Alves Almeida, Beatriz Moraes Martinelli, Maria Carmen Gonçalves Lopes Fernandes, Stephany Cares Hubers, Monique R. M. Ferreira, Daniel Dias Ribeiro, Júlio César Teixeira, José Barreto Campello Carvalheira, Carmen Silvia Passos Lima, Nelson Adami Andreollo, Maurício Etchebehere, Lair Zambon, Ubirajara Ferreira, Alfio José Tincani, Antônio Santos Martins, Cláudio Saddy Rodrigues Coy, José Cláudio Teixeira Seabra, Ricardo Kalaf Mussi, Helder Tedeschi, Joyce Maria Anninchino-Bizzacchi, ADVENTH Cancer Group

**Affiliations:** 1 School of Medical Science, FCM-UNICAMP, University of Campinas, Campinas 13083-888, SP, Braziljoyce@unicamp.br (J.M.A.-B.); 2 Laboratory of Hemostasis, Hematology and Transfusion Medicine Center, Hemocentro—UNICAMP, Campinas 13083-878, SP, Brazil; silmara@unicamp.br (S.A.L.M.);; 3 Clinical Hospital of UFMG, Belo Horizonte 30130-100, MG, Brazil; 4 Department of Obstetrics and Gynecology, Division of Oncology, Women’s Hospital, CAISM-UNICAMP, University of Campinas, Campinas 13083-881, SP, Brazil; 5 Clinical Hospital of Unicamp, Campinas 13083-888, SP, Brazil

**Keywords:** fibrinolysis, plasmin, thrombin, cancer, mortality, STP

## Abstract

**Simple Summary:**

Cancer is a leading cause of death before age 70 and has significant impacts on public health. Cancer patients are at risk for blood clots, tumor progression, and death. The fibrinolytic system, which is involved in blood clotting, shows cooperative effects that facilitate the growth of tumors and the appearance of metastases. This prospective study evaluates the fibrinolytic potential in cancer patients and its association with mortality outcomes. Cancer patients were found to have a hypercoagulability profile and increased plasmin production compared to healthy individuals. Two thrombin generation parameters, maximum amplitude and area under the curve, showed an association with mortality risk. In conclusion, measuring thrombin concentration may help identify cancer patients with an elevated risk of death and further studies can play a crucial role in shedding light on the findings presented here.

**Abstract:**

Cancer is a leading cause of death, and the fibrinolytic system shows cooperative effects that facilitate the growth of tumors and the appearance of metastases. This prospective study aimed to evaluate the fibrinolytic potential in cancer patients and its association with mortality outcomes using the fluorometric method of simultaneous thrombin and plasmin generation. The study included 323 cancer patients and 148 healthy individuals. During the 12-month follow-up, 68 patients died. Compared to the control group, cancer patients showed alterations in thrombin production consistent with a hypercoagulability profile, and an increase in plasmin generation. Mortality risk was associated with two parameters of thrombin in both univariate and multivariable analysis: maximum amplitude (Wald 11.78, *p* < 0.001) and area under the curve (Wald 8.0, *p* < 0.005), while such associations were not observed for plasmin. In conclusion, this was the first study able to demonstrate the simultaneous evaluation of thrombin and plasmin generation in newly diagnosed untreated cancer patients. Patients with cancer have been observed to exhibit a hypercoagulable profile. During the study, two parameters linked to thrombin generation, MA and AUC, were identified and found to have a potential association with mortality risk. However, no associations were found with parameters related to plasmin generation.

## 1. Introduction

Cancer is a significant public health concern and ranks among the top four causes of death before the age of 70. Recent estimations suggest that in 2018, there were 18 million new cases of cancer and 9.6 million deaths [1]. Cancer patients are at increased risk of thromboembolic events, complications, tumor progression, and death [2].

Abnormalities in the hemostatic system, such as disseminated intravascular coagulation, hemorrhagic events, venous thrombosis, and migratory thrombophlebitis, are common disorders found in cancer patients [3]. Platelets, coagulation factors, and the fibrinolytic system exhibit cooperative effects that facilitate tumor growth and the appearance of metastases [4].

The fibrinolytic process depends primarily on the proteolytic activity of serine protease plasmin, which is formed from plasminogen, an inactive zymogen form [5]. Conversion of plasminogen into plasmin can occur through tissue plasminogen activator (tPA) or urokinase-type plasminogen activator (uPA) [6]. The system is regulated by several types of inhibitor, with plasmin being directly inhibited by α2-antiplasmin and α2-macroglobulin, or indirectly by PAI-1 and thrombin-activatable fibrinolysis inhibitor (TAFI). While PAI-1 acts on tPA, TAFI degrades lysine residues from the fibrin surface, reducing plasminogen-tPA complex binding sites and plasmin generation [7,8,9].

The fibrinolytic system is involved in the physiology and pathology of fibrinolysis and hemostasis, in the degradation of the extracellular matrix (ECM), cell migration, tissue remodeling, wound healing, angiogenesis, and inflammation [10]. One of the roles of the fibrinolytic system is its association with clinical progression in cancer patients.

Plasmin is associated with tumor progression due to the degradation of components of the ECM and the basement membrane. Plasmin also plays a role in the activation of matrix metalloproteinases (MMPs), growth factors, and proteolysis of membrane glycoproteins [4,10,11].

Control of ECM degradation allows tumor cells to invade surrounding tissues and access to the bloodstream [12]. Additionally, the activation of MMPs contributes to tumor growth by activating PAR-1 and by potentiating the release of latent forms of growth factors, such as epidermal growth factor (EGF) and transforming growth factor beta (TGF-β) [13]. The fibrinolytic system also shows a proteolytic action on the tissue inhibitor of MMPs, preventing its inactivation [5].

Plasmin regulates tumor nutrition and oxygenation via its ability to promote the formation of blood vessels [11,14]. The release of factors related to tumor growth and a pro-inflammatory environment facilitate angiogenesis and prevent the action of antineoplastic therapies [15]. The fibrinolytic system is intrinsically connected to the coagulation process and has a role in the occurrence of venous thromboembolism (VTE) and hemorrhage events [16].

Prospective data on the fibrinolytic system and its clinical outcomes in cancer patients are scarce [2,17]. To date, there is no “gold standard” test used to evaluate fibrinolytic activity in blood [6]. The available techniques are difficult, time-consuming, and hard to automate [18]. Moreover, the methods used are not sufficiently standardized, making them incomparable to the tests used to evaluate the coagulation process [19].

Global assays have been a promising alternative. The simultaneous thrombin and plasmin (STP) generation assay, like turbidimetric tests, involves the use of coagulation and fibrinolytic activators, with the addition of fluorometric substrates of thrombin and plasmin, allowing the evaluation of single enzyme production [20].

Although the STP assay can sensitively and accurately detect changes in thrombin and plasmin formation associated with hemostatic disorders [20], cancer patients have not been evaluated using the STP assay. Therefore, further tests are needed to assess if STP assay is effective in this scenario. The aim of this study is to evaluate, using a fluorometric global assay, the fibrinolytic potential and its association with the risk of mortality for cancer patients.

## 2. Materials and Methods

### 2.1. Study Group

This prospective multicentric collaborative study was approved by the ethics committee (CEP: 3.441.521) and informed consent was obtained from all participants of the study. Patients with cancer were invited to participate, and those who agreed had blood samples collected and provided information about their medical history by answering a questionnaire. The patients were followed for 6 consecutive months, and a final evaluation was performed after 12 months to verify the occurrence of death.

The inclusion criteria were patients older than 18 years old with a recent cancer diagnosis or progression who agreed to donate a blood sample. The exclusion criteria were patients with leukemia, subjects receiving anticoagulant therapy, patients who had undergone chemotherapy in the last three months or surgery in the last 15 days, and positive history of previous thromboembolic event.

The control group consisted of volunteers aged 18 years old and over with a negative history of cancer or thromboembolic event, who were not receiving anticoagulant treatment and had not undergone surgery in the last 15 days. The volunteers answered a questionnaire about their medical history and a blood sample was collected when they were included in the study.

### 2.2. Simultaneous Thrombin and Plasmin Generation Assay

The blood samples were collected in sodium citrate tubes (3.2%) and centrifuged at 3500 rpm for 15 min at room temperature, and the resulting plasma was centrifugated again under the same conditions to obtain the platelet-poor plasma (PPP). Samples were aliquoted and stored at −80 °C until the tests were carried out.

The STP assay was established according to the protocol described by Simpson et al. (2011) [20] and adapted following the guidelines of Pieters et al. (2018) [21]. To perform the assay, frozen PPP was thawed in a water bath at 37 °C for 3 min. A stock solution of tris-buffered saline (TBS) was prepared, containing 66 mM of Tris and 130 mM NaCl. For each test, a reagent solution was prepared by adding 34 mM of CaCl_2_, 10 pM of tissue factor (lipidated recombinant human tissue factor—Innovin, Siemens Healthcare, Erlangen, Germany) and 900 ng/mL of tPA (recombinant two-chain tissue plasminogen activator—Actylise, Boehringer Ingelheim, Biberach an der Riss, Germany) to the TBS solution. The final concentration of reactants in plasma were 5 pM TF and 450 ng/mL tPA.

Two substrates were prepared with a final concentration of 100 µM and used for the detection of the enzymes, α-thrombin (BOC-VAL-PrO-ARG-MCA, Peptides International, 5 mg) and plasmin (BOC-GLU-LYS-LYS-MCA, Peptides International, 5 mg). The first two wells of the plate (Grelner, 96w Flat bottom, Black Clear) were used as a blank, and the samples were run in duplicate in parallel rows for each substrate, avoiding potential interference and/or interaction in signal detection.

Substrate solutions (20 µL) were added to the wells of the plate, followed by 90 µL of the samples and blank (TBS solution). Next, using a multi-tip automated pipette, 90 µL of the pre-warmed reaction solution (37 °C for 3 min) was added to each well of the plate. Finally, the plate was analyzed in the equipment (Fluoroskan Ascent 2.6—Thermo Fisher Scientific, Vantaa, Finland) and read at a wavelength of 340 nm excitation and 450 nm emission for 4 h at a 45 s intervals time.

**Figure 1:**
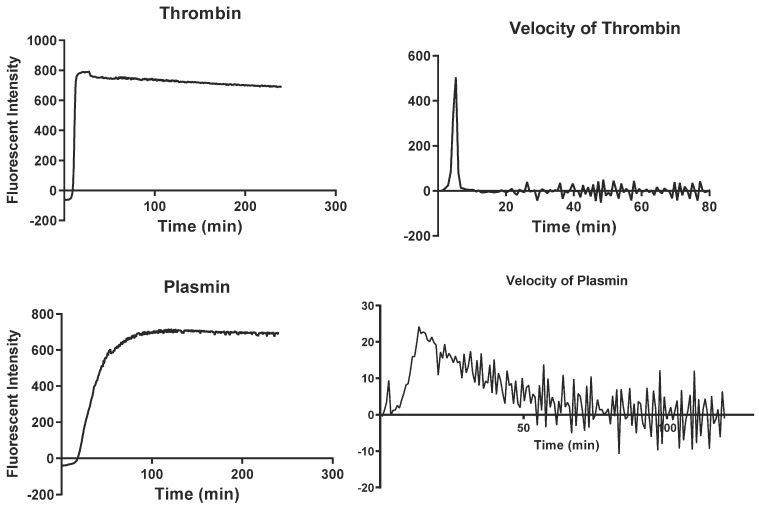
Thrombin and plasmin generation curves obtained after fluorescence reading using STP methodology.

Data analysis was performed using Microsoft^®^ Excel^®^ software version 2307. The curves for thrombin and plasmin were generated by calculating the average at each time point for the duplicated plasma wells, subtracting the reading values of the blank (for thrombin and plasmin separately) (Figure 1). The following parameters were extracted from the curves: (1) Lag time—time when the production of plasmin and thrombin starts, (2) MA—maximum amplitude, (3) tMA—Time to maximum amplitude, (4) AUC—area under the curve. In addition, (5) Vmax—maximum substrate production speed and (6) tVmax—time to reach Vmax were extracted from calculations of the first derivative (velocity). All parameters are reported as a percentage of normal control values used on the plates.

### 2.3. Statistical Analysis

Statistical analyses were performed using GraphPad Prism version 5.0.0 for Windows (GraphPad Software, San Diego, CA, USA) and IBM SPSS Statistics (version 29.0). Continuous variables are reported as median values (25th and 75th percentiles) and categorical variables are reported as absolute frequency and percentage (%). Analysis between groups was performed using the Mann–Whitney test, Fisher’s exact test, or the chi-squared test. Risk of death was assessed using the Kaplan–Meier method, and mortality rates were compared using the long-rank test, in which the variables were dichotomized at the 75th percentile; in other words, the variables are split into two groups based on whether they were above or below the 75th percentile. Cox regression models were used for uni- and multivariable time-to-event (death).

## 3. Results

### 3.1. Cancer Group vs. Control Group

In the period between March 2019 and March 2021, this study enrolled 323 patients with a recent diagnosis of cancer or progression who had not received any previous treatment in the last three months, and 148 controls. Among the cancer patients, 277 were newly diagnosed cases (86%) and 46 had cancer recurrence (14%). Most patients were enrolled within three months of diagnosis (73%).

The age of patients included in this study ranged from 22 to 93 years, with a median of 63 years. The median age for controls was 50 years old. Approximately 51% and 62% of subjects in the cancer and control groups, respectively, were females (Table 1).

Patients with cancer had a higher frequency of smoking habits (41.2%) and alcohol consumption (13.0%). Furthermore, they more frequently used medications for blood pressure control (41.8%) and statin-based medications (17.3%) (Table 1).

The most frequent tumor sites were breast (14.86%), lymphatic system (14.24%), colon/rectum (11.76%), and stomach (7.74%). The majority of patients included in this study were classified in stage III (29.7%). Additionally, subjects of the cancer group showed increased values for WBC (*p* < 0.0001) and platelets (*p* = 0.0001) and decreased values for hemoglobin (*p* < 0.0001), as shown in Table 1.

Cancer patients showed alterations in all parameters evaluated in the thrombin curve (Table 2). These alterations are related to a hypercoagulability profile, evidenced by a higher MA when compared to controls (*p* < 0.0001). In addition, the curve peak is reached earlier (*p* < 0.0001), resulting in a larger area under the curve (*p* < 0.0001). Furthermore, thrombin generation also starts earlier, as can be observed from a shorter lag time (*p* < 0.0001).

When fibrinolytic potential was evaluated, we identified that patients with cancer presented an increase in several parameters associated with plasmin generation (Table 2), that are evidenced by higher MA (*p* < 0.0001), an increase in the area under the curve (*p* < 0.0001), and shorter lag time (*p* < 0.0001). The parameter of lag time shows that plasmin formation starts earlier in patients than in controls. In addition, an increase in the velocity of plasmin production was observed (*p* < 0.0001).

### 3.2. Non-Death Cancer Group vs. Death Cancer Group

During the 12-month follow-up, 68 patients with cancer progressed to death and 10 patients were lost to follow-up. The number of deaths was higher in the first three months (38.24%) when compared with the last period of the follow-up. Furthermore, 10 patients were lost to follow-up (3.1%), 57 (18.2%) subjects underwent surgical procedure, and 96 (30.7%) were hospitalized at least once for more than 3 days. Regarding treatment, 69.0% of recruited patients started chemotherapy and 23.6% underwent treatment with radiotherapy.

Patients who died were at a more advanced age (65 years, *p* = 0.0053); the majority were male (67.6%, *p* = 0.0006) and had a lower body mass index (BMI), with a median of 23 Kg/m^2^ (*p* = 0.0004), and smoking habits were identified in 63.2% of the subjects. The most reported comorbidities in patients who died during the follow-up were hypertension (45.6%) and diabetes (17.6%). This group also showed a significant difference in comorbidities associated with heart disease (*p* = 0.0320) and nephropathies (*p* = 0.0258) (Table 3).

Furthermore, the mortality group showed higher levels of D-dimer when compared to the non-mortality group (*p* = 0.0010). Meanwhile, evaluation of the p-selectin test showed no statistically significant difference between the groups (*p* = 0.2903) (Table 3).

The mortality group showed a higher frequency of subjects classified as stage IV (57.4%, *p* < 0.0001), and the most frequent chemotherapy protocols were those using platinum bases or gemcitabine drugs (61.9%, *p* = 0.0381). Patients who are deceased were classified into four groups according to the cause of mortality: (1) disease progression; (2) infections/complications; (3) thromboembolic complications; and (4) unclear. The most frequent cause of death was disease progression (41.2%) (Table 3).

In the group that progressed to death, the most common types of cancer were stomach (17.6%), lymphoma (13.2%), and lung/pleura (11.8%). However, when the lethality percentage of each site was evaluated, brain (67%), pancreas (60%), and gastric cancer (52%) were the most frequent (see Appendix A).

We observed that patients who died showed an increased pattern for thrombin generation, as evidenced by higher MA (*p* = 0.001) and AUC of thrombin (*p* = 0.018), associated with a lower fibrin destruction rate, as shown by a decrease in the speed of plasmin production—Vmax (*p* = 0.016) (Table 4).

**Figure 2:**
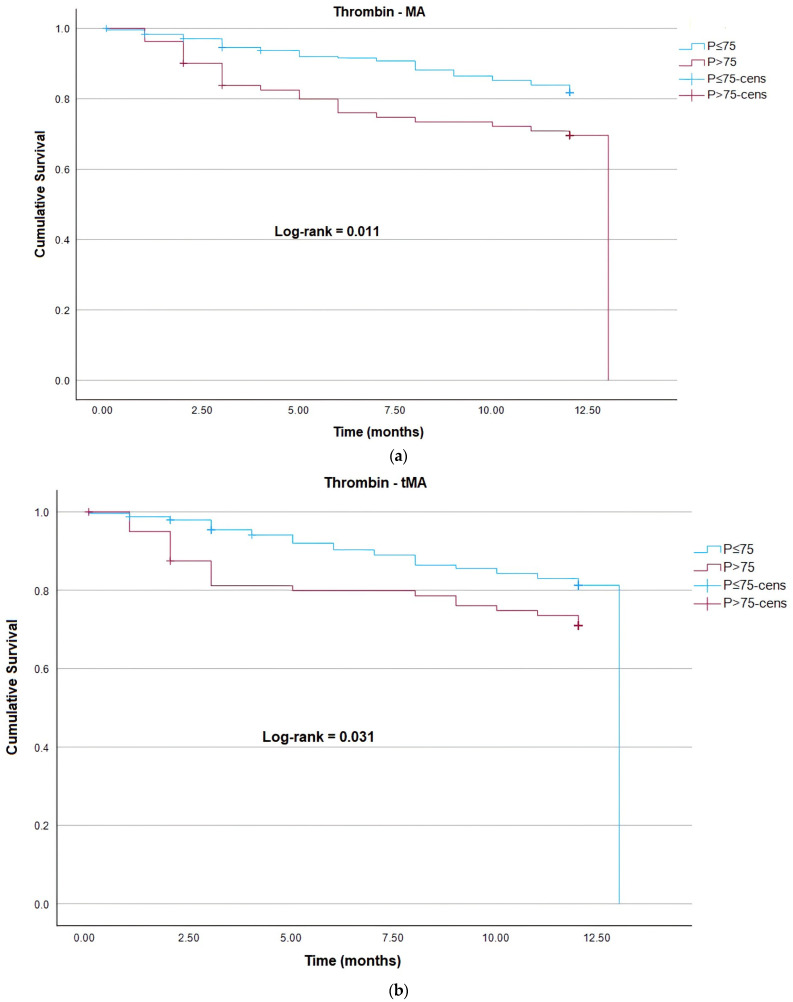

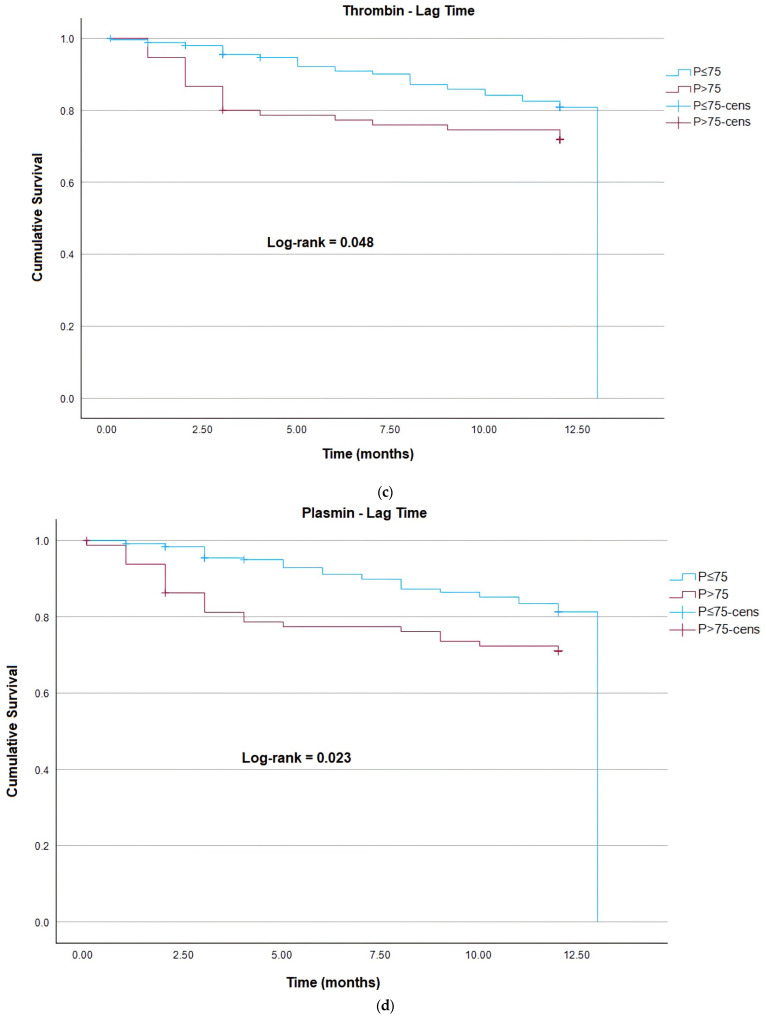

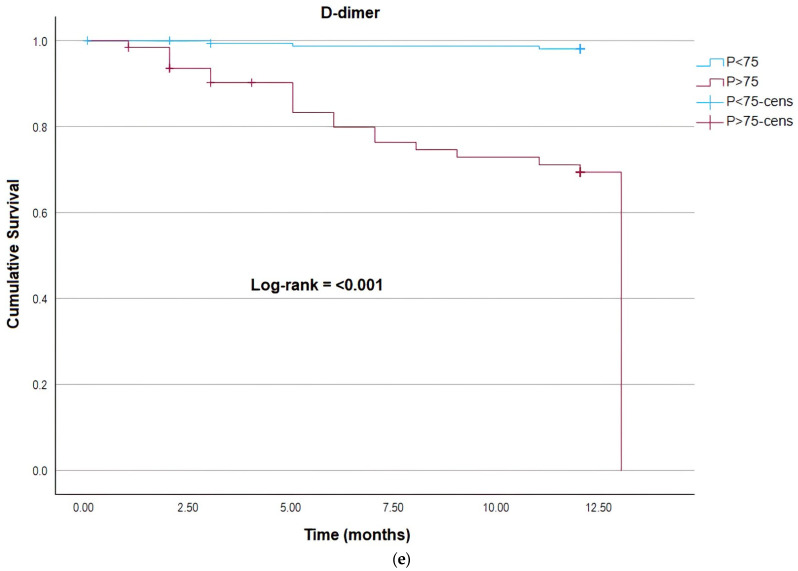
Kaplan–Meier analysis for risk of death for the parameters of (**a**) maximum amplitude of thrombin; (**b**) time to maximum amplitude of thrombin, (**c**) thrombin lag time, (**d**) plasmin lag time, and (**e**) D-dimer. Blue line—patients below the 75th percentile (P < 75), and purple line—patients above the 75th percentile (P > 75). *p*-value from the log-rank analysis, +Cens—Censured: patients who were lost to follow-up.

For evaluation of mortality risk using the Kaplan–Meier method, the parameters of plasmin generation and thrombin were dichotomized into two groups using the 75th percentile. Statistically significant values (log-rank *p* < 0.05) were identified for the parameters of thrombin MA, thrombin tMA, thrombin lag time, and plasmin lag time above the 75th percentile, meaning that patients in this groups have a higher risk of mortality (Figure 2a–d). Similarly, a significative association between D-dimer and risk of death was also identified for patients above the third quartile (Figure 2e).

However, when regression analysis using the Cox univariate test was performed, only parameters associated with thrombin maximum amplitude (*p* < 0.001), thrombin area under the curve (*p* = 0.005), and time for maximum velocity of plasmin (*p* = 0.027) showed an association with mortality risk (Table 5). The association was maintained for MA (*p* = 0.004) and AUC (*p* = 0.025) of thrombin after adjustment for demographic and clinical data (Table 6) previously associated with the risk of mortality (see Appendix B and C), whereas for plasmin, these associations were not maintained. Thereafter, parameters related to plasmin generation did not interfere with the risk of mortality.

## 4. Discussion

This was the first prospective study to evaluate simultaneous thrombin and plasmin generation using fluorometric substrates in patients recently diagnosed with cancer.

The study showed that cancer patients had alterations in all coagulation parameters compared to controls, including a hypercoagulability profile, as evidenced by an increase in parameters related to thrombin generation, as well as higher amplitudes and speed of plasmin generation. Parameters related to AUC and amplitude of thrombin showed an association with the risk of mortality, but this association was not observed regarding plasmin parameters.

The relationship between malignancy and hemostatic disorders is well-established in the literature, as tumor cells have the ability to alter the components of the hemostasis process [22,23]. The hypercoagulability profile found in this study partially corroborates with the work of Gronostaj et al. (2013) [24] and Posch et al. (2019) [2], both of whom used turbidimetric methods to evaluate the coagulation and lysis process in cancer patients. Similar to our findings, Gronostaj also described a faster clot formation rate, as indicated by a shorter lag phase, but did not find higher amplitudes, while Posch observed elevated peak absorbance and amplitude but a longer lag phase.

In comparison to controls, an increase in parameters related to plasmin production (amplitude and speed) was observed. Such an increase in fibrinolytic potential has also been observed in studies related to patients with leukemia, as described in the review by Kwaan (2019) [25] and Hoffman (2001) [26]. This was also described in patients with ovarian cancer compared to patients with benign cysts, as shown in the study by KOH et al. (2001) [27], who observed hypercoagulability, seen in increased F1 + 2 and decreased antithrombin, as well as increased fibrinolysis, indicated by elevated D-dimer levels.

When evaluating patients who progressed to death, an increased formation of blood clots was evidenced by higher MA and AUC values for thrombin generation, as well as a lower fibrin destruction rate indicated by a decrease in the velocity of plasmin production, which is associated with a shorter time to achieve this speed.

We found that plasmin parameters were not related to mortality in Cox multivariate analysis, as observed by Posch et al. (2019) [2], who found that CLT was statistically significant in the univariable Cox regression, but not in the Kaplan–Meier analysis with a cut-off at the 75th percentile and not after multivariate regression adjustments.

Regarding the results involving thrombin generation parameters as possible risk markers for mortality, after adjustments for clinical and demographic parameters, it was observed that thrombin MA and thrombin AUC are important predictors, especially the maximum amplitude.

Altered clot formation as a predictor of mortality was also verified in the study by Posch and colleagues (2019) [2], who proposed peak absorbance as a biomarker for ATE and death in the cancer population, and in the work of Giaccherini et al. (2022) [28], who described two markers, D-dimer and TG-ETP (thrombin generation—endogenous thrombin potential), as predictors for mortality using the thrombogram method. The link between thrombin generation and cancer mortality may be associated with its potential in the process of angiogenesis and tumor metastasis, leading to disease progression [29,30,31,32,33].

Furthermore, the Kaplan–Meier analysis indicated the potential predictor of D-dimer above the 75th percentile for cancer mortality risk, corroborating with several studies that have already established such an association [28,33,34,35].

We emphasize that, unlike the findings observed in our study regarding the fibrinolytic potential, Gronastaj et al. (2013) [24] and Posch et al. (2019) [2] found that cancer patients had impaired fibrinolysis, which could be explained by the fact that they did not evaluate individual plasmin production and investigated fibrinolysis as a measure of time (CLT—clot lysis time).

These findings could be explained by the work of Undas et al. (2014) [36], Gronastaj et al. (2013) [24], and Geffen et al. (2011) [37], who reported in their studies that an increase in thrombin generation adversely affects the properties of the plasma clot, resulting in a thinner fibrin network and increasing resistance to fibrinolysis. In other words, increased thrombin generation can result in impaired fibrinolysis, as the fiber arrangement affects tPA bonds and the fibrinolysis rate.

Kvolik et al. (2010) [23] explained that the global fibrinolytic capacity may be frequently increased in cancer patients because the fibrinolytic system is activated during tumor growth to solubilize the fibrin clot, which may prove a potential compensatory balancing process in order to achieve hemostasis. The dysregulation of the components of the fibrinolytic system was also described by Kwaan and Lindholm (2019) [38] in malignancy, and explained by involvement not only in the hemostatic process, but also linked to involvement in tumor metastasis and angiogenesis.

This compensatory profile was also explained by Hyman, Soff, and Kampel (2011) [39] in patients with DIC in solid cancers who presented markers of increased thrombin generation, such as the TAT complex, as well as DD levels, showing the degree of fibrinolysis. However, fibrinogen levels when measured are usually normal, indicating a compensated fibrinolysis.

The study by Collet and colleagues (2000) [40] showed that tight clots made of fine fibers lysed slower, even with a faster cleavage velocity than thick and loose fibers. This fact can be explained by the architecture of the fibrin network being more important than the fiber diameter in regulating the distribution of fibrinolytic components during the fibrinolysis process. Thus, the increased profile and high plasmin generation speed in the oncological group may be associated with a compensatory profile associated with fibrinolysis resistance due to fibrin network conformation.

## 5. Conclusions

This was the first prospective study able to demonstrate the simultaneous evaluation of thrombin and plasmin generation in newly diagnosed untreated cancer patients. We observed a hypercoagulability profile in cancer patients associated with higher amplitude and velocity of plasmin production. Additionally, we identified two parameters with a potential association with mortality risk: MA (maximum amplitude) and AUC (area under the curve) for thrombin generation. However, no association between parameters of plasmin and mortality was found. Conducting a more in-depth evaluation of the parameters involved in thrombin and plasmin generation among the oncological population can provide valuable insights into mortality risk. By exploring these aspects, we can better understand the underlying mechanisms and potentially develop more targeted interventions to improve patient outcomes.

## Figures and Tables

**Table 1:** Comparison of clinical and laboratory data between cancer patients and control group.

Variable	Cancer Patients (N = 323)	Control Group (N = 148)	*p*-Value
Age (years)	63 (52–71)	52 (40–60)	<0.0001 ^1^
Male gender	159 (49.2%)	56 (37.8%)	0.022 ^2^
Female gender	164 (50.8%)	92 (62.2%)	
BMI (Kg/m^2^)	25.1 (21.9–29.3) N = 319	28.0 (25.1–31.2) N = 144	<0.0001 ^1^
Smoker or ex-smoker	133 (41.2%)	27 (18.4%) N = 147	<0.0001 ^2^
Alcoholism or ex-alcoholism	42 (13.0%)	2 (1.4%)	<0.0001 ^2^
Medications	/	/	/
Antihypertensive	135 (41.8%)	41 (27.7%)	0.004 ^2^
Statin	56 (17.3%)	8 (5.4%)	0.0003 ^2^
Diabetes medications	45 (13.9%)	14 (9.5%)	0.2298 ^2^
AAS	21 (6.5%)	4 (2.7%)	0.1198 ^2^
Tumor site	/	/	/
Bladder	04 (1.24%)	-	-
Brain	06 (1.86%)	-	-
Breast	48 (14.86%)	-	-
Colon and Rectum	38 (11.76%)	-	-
Esophagus	13 (4.02%)	-	-
Kidney	07 (2.17%)	-	-
Liver and bile duct	12 (3.72%)	-	-
Lung	21 (6.50%)	-	-
Lymphoma	46 (14.24%)	-	-
Myeloma	18 (5.57%)	-	-
Other sites	65 (20.12%)	-	-
Ovary	04 (1.24%)	-	-
Pancreas	06 1.86%)	-	-
Stomach	25 (7.74%)	-	-
Uterus	10 (3.10%)	-	-
Cancer stage	/	/	/
Stage I	47 (14.55%)	-	-
Stage II	86 (26.63%)	-	-
Stage III	101 (31.27%)	-	-
Stage IV	89 (27.55%)	-	-
WBC (10^3^/µL)	7.5 (5.8–9.5) N = 315	6.1 (5.3–7.2) N = 146	<0.0001 ^1^
Hemoglobin (g/dL)	12.8 (10.8–13.9) N = 315	13.8 (13.0–15.1) N = 146	<0.0001 ^1^
Platelets (10^3^/µL)	269 (204–344) N = 315	241 (208–271) N = 146	0.0001 ^1^

^1^—Mann–Whitney test, ^2^—Fisher’s exact test.

**Table 2:** Comparison of the results of the parameters of STP test between control group and cancer group. *p*-value: Mann–Whitney test.

Parameters	Control Group N = 148	Cancer Group N = 323	*p*-Value
Plasmin (%NC)	/	/	/
MA	102.8 (92.4–110.9)	122.9 (112.1–135.7)	<0.0001
T(MA)	110.2 (97.9–136.9)	107.9 (96.4–133.1)	0.518
Lag Time)	150.0 (133.3–166.6)	125.0 (107.1–146.6)	<0.0001
AUC	90.7 (76.9–103.1)	111.7 (99.8–127.5)	<0.0001
Vmax	109.4 (86.5–159.8)	174.6 (118.9–179.8)	<0.0001
T(Vmax)	319.4 (195.7–911.5)	250.0 (140.9–686.4)	0.007
Thrombin (%NC)	/	/	/
MA	102.4 (93.6–110.7)	112.7 (104.3–123.3)	<0.0001
T(MA)	166.6 (139.1–204.4)	143.7 (118.2–177.3)	<0.0001
Lag Time	140.0 (120.0–160.0)	115.4 (88.9–150.0)	<0.0001
AUC	101.4 (92.5–113.1)	119.1 (109.2–130.9)	<0.0001
Vmax	79.1 (60.5–110.0)	97.5 (68.7–131.7)	0.0003
T(Vmax)	140.0 (120.8–160.0)	114.3 (90.9–145.4)	<0.0001

**Table 3:** Comparison of clinical and laboratory data among patients with and without progression to death.

Variable	DEATH		*p*-Value
	No N = 245	Yes N = 68	
Age (years)	62.0 (51.0–70.0)	65.0 (59.0–73.0)	0.0053 ^1^
Gender	/	/	/
Male	107 (43.7%)	46 (67.6%)	0.0006 ^2^
Female	138 (56.3%)	22 (32.4%)	
BMI (Kg/m^2^)	25.6 (22.8–29.7) N = 243	23.0 (20.3–27.5) N = 66	0.0004 ^1^
Comorbidities	/	/	/
Hypertension	103 (42.0%)	31 (45.6%)	0.6780 ^2^
Diabetes	38 (15.5%)	12 (17.6%)	0.7089 ^2^
Previous thrombosis	16 (6.5%)	6 (8.8%)	0.5909 ^2^
Lung disease	12 (4.9%)	3 (4.4%)	1.0000 ^2^
Heart disease	13 (5.3%)	9 (13.2%)	0.0320 ^2^
Nephropathy	4 (1.6%)	5 (7.4%)	0.0258 ^2^
Habits	/	/	/
Smoking/ex-smoker	87 (35.5%)	43 (63.2%)	<0.0001 ^2^
Alcoholic/ex-alcoholic	33 (13.5%)	8 (11.8%)	0.8402 ^2^
Laboratory tests	/	/	/
D-dimer (ng/mL)	493 (290.5–1018) N = 193	882 (421–2026) N = 48	0.0010 ^1^
P-selectin (ng/mL)	76.3 (48.9–98.9) N = 174	82.9 (54.9–122.9) N = 52	0.2903 ^1^
Cancer stage	/	/	/
I	38 (16.0%)	7 (10.3%)	<0.0001 ^3^
II	79 (32.0%)	2 (2.9%)	
III	79 (32.0%)	20 (29.4%)	
IV	49 (20.0%)	39 (57.4%)	
Cause of death	/	/	/
Disease progression	/	28 (41.2%)	/
infections/complications	/	23 (33.8%)	/
thromboembolic complications	/	5 (7.4%)	/
Unclear	/	12 (17.6%)	/
Chemotherapy protocol	/	/	/
Platinum and/or gemcitabine-based chemotherapy	75 (43.1%) N = 174	26 (61.9%) N = 42	0.0381 ^2^
Without platinum and/or gemcitabine-based chemotherapy	99 (56.9%) N = 174	16 (38.1%) N = 42	

^1^—Mann–Whitney test, ^2^—Fisher’s exact test, ^3^—Chi-square test.

**Table 4:** Comparison of the results of the parameters of STP test between non-death group and death group. *p*-value: Mann–Whitney test.

Parameters	DEATH		*p*-Value
	No N = 245	Yes N = 68	
Plasmin	/	/	/
MA	122.8 (111.7–135.7)	124.1 (115.6–137.5)	0.086
T(MA)	107.9 (96.2–132.5)	111.2 (96.9–146.4)	0.639
Lag Time	123.1 (106.2–143.7)	133.3 (114.5–153.3)	0.067
AUC	111.6 (99.3–127.5)	111.9 (101.5–127.6)	0.673
Vmax	148.2 (122.1–183.1)	129.3 (109.5–173.3)	0.016
T(Vmax)	282.6 (143.5–795.8)	178.0 (135.6–342.3)	0.016
Thrombin	/	/	/
MA	111.8 (103.5–121.9)	117.7 (108.6–130.0)	0.001
T(MA)	142.8 (116.7–171.4)	146.0 (123.9–204.8)	0.138
Lag Time	115.4 (91.7–150.0)	113.9 (88.9–161.9)	0.613
AUC	117.6 (108.5–130.5)	123.9 (110.9–133.0)	0.018
Vmax	95.2 (68.8–130.2)	107.1 (68.9–140.3)	0.359
T(Vmax)	114.3 (90.9–144.4)	114.3 (89.8–156.5)	0.797

**Table 5:** Univariable Cox proportional hazards regression. N = 323. Endpoint: death. HR: hazard ratios—relative risk of the event between the two groups. 95%IC: 95% confidence interval. Wald test: A statistical significance test for individual coefficients, used to determine the impact of a variable on the outcome in a regression model.

Dependent Variable	Explanatory Variable	HR	95%IC	*p*-Value	Wald
DEATH	Thrombin MA	1.02	1.01–1.04	<0.001	11.78
	Thrombin tMA	1.00	0.99–1.01	0.107	2.59
	Thrombin Lag Time	1.00	0.99–1.01	0.266	1.24
	Thrombin AUC	1.02	1.01–1.03	0.005	8.00
	Thrombin Vmax	1.00	0.99–1.01	0.392	0.73
	Thrombin tVmax	1.00	0.99–1.01	0.478	0.50
	Plasmin MA	1.01	0.99–1.02	0.101	2.69
	Plasmin tMA	1.01	0.99–1.01	0.408	0.69
	Plasmin Lag Time	1.01	1.00–1.01	0.065	3.41
	Plasmin AUC	1.01	0.99–1.01	0.721	0.13
	Plasmin Vmax	1.00	0.99–1.01	0.614	0.25
	Plasmin tVmax	1.00	0.99–1.00	0.027	4.92

**Table 6:** Multivariable Cox proportional hazards regression. N = 319. Endpoint: death (n = 66). HR: hazard ratios—relative risk of the event between the two groups. 95%IC: 95% confidence interval. Ref—reference. Model 1: Evaluate the parameter AUC of thrombin after adjustments for clinical and demographic data. Model 2: Evaluate the parameter MA of thrombin after adjustments for clinical and demographic data. Model 3: Evaluate the parameter tVmax of plasmin after adjustments for clinical and demographic data. High risk site-based mortality according to the survival rate described in SEER: pancreas, esophagus, liver and intrahepatic bile duct, and lung.

Model	Variable	HR (95% IC)	*p*-Value
Model 1—Death	Male gender	1.78 (1.04;3.06)	0.037
	BMI (Kg/m^2^)	0.95 (0.90;1.00)	0.040
	Smoking/ex-smoker	1.99 (1.15;3.45)	0.014
	High risk tumor	2.00 (1.15;3.47)	0.014
	Cancer stage I	Ref.	<0.001
	Cancer stage II	0.14 (0.29;0.67)	0.014
	Cancer stage III	0.93 (0.39;2.24)	0.868
	Cancer stage IV	2.25 (0.97;5.19)	0.058
	Thrombin AUC	1.02 (1.01;1.03)	0.025
Model 2—Death	Male gender	1.86 (1.08;3.21)	0.025
	BMI (Kg/m^2^)	0.95 (0.90;1.00)	0.046
	Smoking/ex-smoker	1.91 (1.10;3.30)	0.021
	High risk tumor	2.11 (1.21;3.69)	0.009
	Cancer stage I	Ref.	<0.001
	Cancer stage II	0.12 (0.25;0.60)	0.009
	Cancer stage III	0.83 (0.34;2.03)	0.689
	Cancer stage IV	2.05 (0.88;4.78)	0.095
	Thrombin MA	1.02 (1.01;1.04)	0.004
Model 3—Death	Male gender	1.58 (0.93;2.71)	0.094
	BMI (Kg/m^2^)	0.95 (0.91;1.00)	0.045
	Smoking/ex-smoker	1.97 (1.15;3.40)	0.014
	High risk tumor	1.67 (0.96;2.89)	0.069
	Cancer stage I	Ref.	<0.001
	Cancer stage II	0.16 (0.32;0.75)	0.021
	Cancer stage III	1.01 (0.42;2.42)	0.990
	Cancer stage IV	2.83 (1.26;6.37)	0.012
	Plasmin tVmax	1.00 (1.00;1.00)	0.167

df = 8 *p* = <0.001.

## Data Availability

The data presented in this study are available on request from the corresponding author. The data are not publicly available due to privacy restrictions.

## Appendix A

**Table A1:** Distribution of patients according to the cancer site and the number of deaths recorded by each type in absolute frequency (n) and percentage (%).

Cancer Site	Cancer (N)	Death (N)	Death (%)
Brain	6	4	66.67
Pancreas	5	3	60.00
Stomach	23	12	52.17
Skin	6	3	50.00
Testis	2	1	50.00
Esophagus	13	6	46.15
Lung	21	8	38.10
Liver	9	3	33.33
Myeloma	15	5	33.33
Intestine	10	3	30.00
Prostate	8	2	25.00
Lymphoma	46	9	19.57
Head and neck	21	4	19.05
Kidney	7	1	14.29
Uterus	10	1	10.00
Rectum	21	2	9.52
Colon	16	1	6.25

## Appendix B

**Table A2:** Multivariable Cox proportional hazards regression. N = 319—Endpoint: Death (n = 66) HR: hazard ratios. 95%IC: 95% confidence interval.

Variable	HR (95% IC)	*p*-Value
Age	1.01 (0.99;1.03)	0.208
Male gender	1.86 (1.07;3.23)	0.027
BMI (Kg/m^2^)	0.93 (0.88;0.98)	0.005
Heart Disease	2.29 (1.08;4.87)	0.031
Nephoropathy	1.61 (0.64;4.10)	0.315
Smoking/ex-smoker	2.19 (1.27;3.76)	0.005

df = 6 *p* = <0.001.

## Appendix C

**Table A3:** Multivariable Cox proportional hazards regression. N = 323—Endpoint: Death. HR: hazard ratios. 95%IC: 95% confidence interval. Ref—reference.

	HR (95% IC)	*p*-Value
Tumor Site—TEV Group of Risk		
High risk tumor	2.81 (1.62;4.87)	<0.001
Tumor site—Mortality group risk		
High risk tumor	2.05 (1.18;3.56)	0.011
Stage		
Cancer stage I	ref	<0.001
Cancer stage II	0.22 (0.05;1.07)	0.061
Cancer stage III	1.91 (0.77;4.77)	0.166
Cancer stage IV	4.48 (1.90;10.57)	<0.001
Chemotherapy protocol		
Without chemeotherapy	ref	0.006
Chemeotherapy based on platin	0.41 (0.23;0.73)	0.002
Chemeotherapy without platin	0.48 (0.25;0.93)	0.030

df = 7 *p* = <0.001.
